# American Medical Association.—Portland

**Published:** 1905-03

**Authors:** 


### American Medical Association.—Portland.

IT HAS been officially announced that this year's meeting of the American Medical Association will be held in July, instead of June, which latter is the usual time when the meeting place is located north of the line of the Ohio river. Portland, Oregon, is the place and July 11-14, is the time for the next meeting.

It is more than probable that this meeting will be more numerously attended than were others that have been held on the Pacific coast. Two reasons, among others, will contribute to increase the attendance in a marked degree: first, the railroads were never able before to accommodate traffic and make tourists as comfortable as now; and, second, the Lewis and Clarke Exposition,—the Western World's Fair,—will furnish an attraction far beyond any heretofore offered on the Pacific slope.

One of the principal points for the visitor to settle is the route by which he will travel, both outward and homeward bound. For Buffalo members and visitors as well for those living eastward on this parallel, Chicago will afford the first objective, which can be reached by several available roads. From there to Portland the natural route is the Northwestern Line to Omaha, and thence by the Union Pacific direct to Portland. Returning, the Northern Pacific Railroad will afford a convenient and delightful homeward trip via Saint Paul, and by the Northwestern thence to Chicago. Individual preferences and other considerations naturally govern the selection of a route, but those indicated, from directness, equipment, comfort and speed, will naturally secure the largest measure of travel, not only from the territory named, but from points westward as far as Omaha, and even beyond that point.

We advise our readers to take up this subject with the agents of the several lines at an early date. By so doing they will obtain advantages that are denied the late applicants. If the railroads ascertain at an early day, even approximately the number for whom provision must be made, they will be enabled to take better care of their passengers. The agents of the Northwestern Line, the Union Pacific, and Northern Pacific railroads are located in the Ellicott Square, Buffalo, and will be glad to furnish information, rates, and other data to applicants.

Inasmuch as the Northwestern is the initial line, perhaps it will simplify matters to communicate direct with the Buffalo office, which is located at 301 Main street; telephone, Seneca 884.

---

JUDGE DAVIS, of Philadelphia, in his charge to the jury in the case of Thomas E. Eldridge, charged with illegally practising medicine, gave an excellent definition of the practice of medicine in the first paragraph of his charge quoted:

> The practice of medicine consists in the offering of service and assuming the responsibilities of treating diseases, deformities and injuries, no matter by what means this is professed to be done.
>
> Every state, because of the inherent nature of the calling of medicine, possesses the right both constitutionally and legally to demand a standard degree of qualification which will protect citizens from the consequences of incompetency and unskilled practice.
>
> Anyone practising medicine without the license of the state, which license is on the part of the state a guarantee of the possession of the qualification to safely pursue medical practice, is an illegal practitioner.
>
> Everyone, therefore, who offers service as outlined, no matter by what means he professes to treat diseases, deformities and injuries, must, as a condition precedent thereto, have obtained the license in accordance with the laws of the commonwealth.

---

DR. THOMAS DARLINGTON, the efficient health commissioner of greater New York, is conducting an active campaign against the spitting nuisance. During one day in every week the police department, at the instigation of Dr. Darlington, stations policemen at theaters, in the subway and other public places, who arrest all spitters. They are taken before a magistrate at the tombs and fined two dollars each for the first offense. The violators of the ordinance are locked up pending trial; hence the punishment seems severe, but no more than it should be when the magnitude of the crime is considered. We hope some day to see Buffalo take a similar stand against this filthy and dangerous habit.

---

THE Kinesipathy bill, the osteopathy bill, and the optometry bill are a fine triad. The proponents of these bills should pool their issues and make a combined attack upon the legislature. They might, with great propriety, invite Antonius, the "Great Cooper" and "Dr." Finch to help them in their nefarious attempts to break down the Medical Practice Act.

